# First biological report on the genus *Cantonius* (Buprestidae, Agrilinae, Aphanisticini), with descriptions of two new species from China

**DOI:** 10.3897/BDJ.11.e98405

**Published:** 2023-03-02

**Authors:** Xiao-Di Shi, Zhi-Ming Wu, Xiao-Hua Dai, Jia-Sheng Xu, Hai-Tian Song

**Affiliations:** 1 Leafminer Group, School of Life Sciences, Gannan Normal University, Ganzhou, China Leafminer Group, School of Life Sciences, Gannan Normal University Ganzhou China; 2 Qiyunshan National Nature Reserve, Ganzhou, China Qiyunshan National Nature Reserve Ganzhou China; 3 Fujian Academy of Foresty, Fuzhou, China Fujian Academy of Foresty Fuzhou China

**Keywords:** host plants, leaf-mines, habitat, biology, Bambusoideae

## Abstract

**Background:**

The genus *Cantonius* Théry, 1929 is a small group with two subgenera and 12 species. However, the biology of this genus is still unknown.

**New information:**

In this paper, three species of the genus *Cantonius* Théry, 1929 were found on bamboo leaves, revealing for the first time that *Cantonius* species are also leaf-miners. Two new species were recorded from Jiangxi Province and are described here: Cantonius (Cantonius) anjiensis **sp. n.** (host plant: *Pleioblastusamarus*) and Cantonius (Procantonius) qiyunensis **sp. n.** (host plant: *Bambusablumeana*) followed by C. (P.) austrisinicus Kalashian, 2021 (host plant: *Oligostachyumpaniculatum*) recorded from Guangxi Province. Including habitats, photos of three species together with C. (P.) qiyunensis **sp. n.** pupa, host plants, and leaf mines of the three species are presented. Moreover, the bionomics and habits of the genus are discussed for the first time, and a hypothesis for the distribution of *Cantonius* is provided.

## Introduction

The genus *Cantonius* Théry, 1929 belongs to the tribe Aphanisticini (Buprestidae, Agrilinae). It is a small group with only 12 known species belonging to two subgenera - the nominotypical subgenus and *Procantonius* Kalashian, 2004. Except for two species recorded from Laos, the remaining ten species are distributed in the subtropical regions of China, namely C. (C.) obenbergeri Gebhardt, 1928, C. (C.) terryi Théry, 1929, C. (C.) klapperichi Obenberger, 1940, C. (C.) szechuanensis Obenberger, 1958, C. (C.) bolmi Kalashian, 2004, C. (C.) fokienicus Kalashian, 2004, C. (C.) nitridifrons Kalashian, 2004, C. (P.) obesus Obenberger, 1940, C. (P.) khnzoriani Kalashian, 2004 and C. (P.) austrisinicus Kalashian, 2021 (Kalashian 2004; Kalashian 2021). In the recent book for Chinese Buprestidae, seven of the ten species are included and colour photographs of the types are shown for the first time (Peng et al. 2021).

Insects that mine leaves may have far-reaching and profound effects on their host plants (Liu et al. 2015). The Family Buprestidae comprises about 21 genera belonging to two subfamilies and four tribes that have been recorded with leaf-mining behaviour (Xiao et al. 2017). Furthermore, large-scale molecular phylogeny has revealed new insights into relationships among Buprestidae taxa as well as multiple evolutionary origins of the buprestid larval leaf-mining habit (Evans et al. 2015). However, *Cantonius* as a group with leaf-mining habit, so far there is no information about the leaf-mining behaviour, their host plants or biology. In this study, description of two new species as well as information about their hosts, leaf-mines, habitat, and biology are provided.

## Materials and methods

Under laboratory conditions, the adults were reared from larvae or pupae in the leaves of their hosts for the following time periods: C. (C.) anjiensis **sp. n.** (from November to March, 2015, 2016 and 2017), C. (P.) qiyunensis **sp. n.** (from January to March, 2021), and C. (P.) austrisinicus Kalashian, 2021 (from January to April, 2015). Host-plant leaves with leaf-mines were scanned by an Epson Expression 10000XL scanner. Adult habitus and genitals were photographed by a Keyence VHX-5000 digital microscope with the Keyence VH-Z20R zoom lens (20–200×). The descriptive terminology of morphological structures and the classification follows Kalashian (2004, 2021).

Abbreviations for collections in this study are:

- **LGNU** Leafminer Group, Gannan Normal University, Ganzhou, China;
- **CHTS** Collection of Hai-Tian Song, Fuzhou, China.

## Taxon treatments

### Cantonius (Cantonius) anjiensis

Shi, Song & Xu sp. nov.

C8BDDDF9-FB3B-5D24-9E79-7E31DFA6BEEE

809DA7B7-A784-4E5A-99E9-FD27361CCF70

#### Materials

- **Type status:** Holotype. **Occurrence:** catalogNumber: SXD2021005♂; sex: male; occurrenceID: 6A87D4D8-594E-56A9-B811-AB6A22B2825E; **Location:** country: China; stateProvince: Jiangxi Province; county: Longnan County; municipality: Ganzhou City; locality: Anji mountains; verbatimElevation: 300–400 m; locationRemarks: label: Anji mountains, Longnan County, Ganzhou City, Jiangxi Province, elevation 300–400 m, feeding larvae in leaf-mines on *Pleioblastusamarus*, adults emerged 12.Ⅲ.2016, Jia-Sheng Xu leg.; **Record Level:** institutionCode: LGNU
- **Type status:** Paratype. **Occurrence:** catalogNumber: SXD2021006♂, SXD2021007♀; sex: 1 male, 1 female; occurrenceID: 646AD091-20F1-5A3F-8791-C85051EB35F3; **Location:** locationRemarks: same label data as holotype; **Record Level:** institutionCode: CHTS
- **Type status:** Paratype. **Occurrence:** catalogNumber: SXD2021004♀; sex: female; occurrenceID: 6AFCCC52-F38D-566A-ABB4-EFB1FAA21048; **Location:** locationRemarks: same label data as holotype, but adults emerged 27.Ⅲ.2017, Wan-Hua Liu Leg.; **Record Level:** institutionCode: LGNU

#### Description

**Figure 1.**
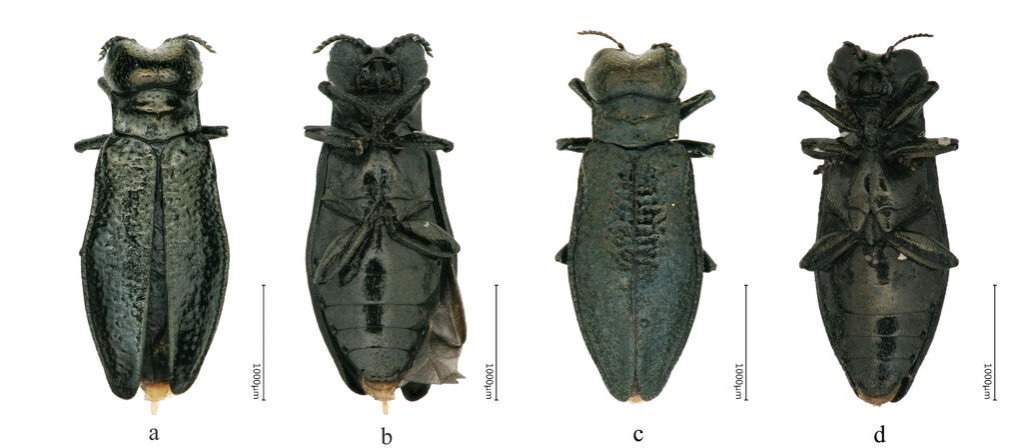
Habitus of Cantonius (Cantonius) anjiensis **sp. n.**: **a** dorsal view of holotype male; **b** ventral view of holotype male; **c** dorsal view of paratype female; **d** ventral view of paratype female.

Body length 3.1–3.4 mm, body width 1.1–1.4mm. Body slender, 2.5–2.7 times as long as wide. Colour black, head, pronotum and elytra with more or less distinct cupreous shine. Surface microreticulated, pronotal convexities and elytral rugae smooth (Fig. 1).

Head extremely large, distinctly larger than pronotum, with sides convex strongly and regularly, widest at level much closer to posterior margins of eyes than to anterior margin of pronotum. Eyes rather small, slightly convex in male, but more convex in female. Male temples are about 1.9 times as long as the longitudinal diameter of the eye in lateral view, whereas female temples are around 2.1 times as long. Forehead depressed widely and deeply. Surface with shallow, small and dense punctures and equally distributed thin pubescence. Antennae with 10 antennomeres; male antennomeres serrate, about twice as wide as long; female antennomeres are approximately equilateral.

Pronotum 1.6–1.8 times as wide as long, much narrower than head and elytra, with anterior margin distinctly bisinuate, posterior margin bisinuate with slightly protruded basal lobe widely cut posteriorly. Anterior margin slightly wider than posterior margin in male, but obviously narrow in female and mid-point much higher than anterior angles. Lateral margin strongly straight. Supralateral carinae starting near basal angles of pronotum, slightly convergent anteriorly, reaching discal convexity. Surface with a few rather large, shallow punctures in depressed portions of disc. Scutellum transversely triangular.

Elytra 1.9–2.0 times as long as wide, widest at middle. Apices rounded and widely separated. Elytral sides emarginated slightly concave in anterior 1/3, with a narrow depression. Surface with irregular, transverse rugae and large, shallow punctures.

Tibiae strongly arched.

**Figure 2.**
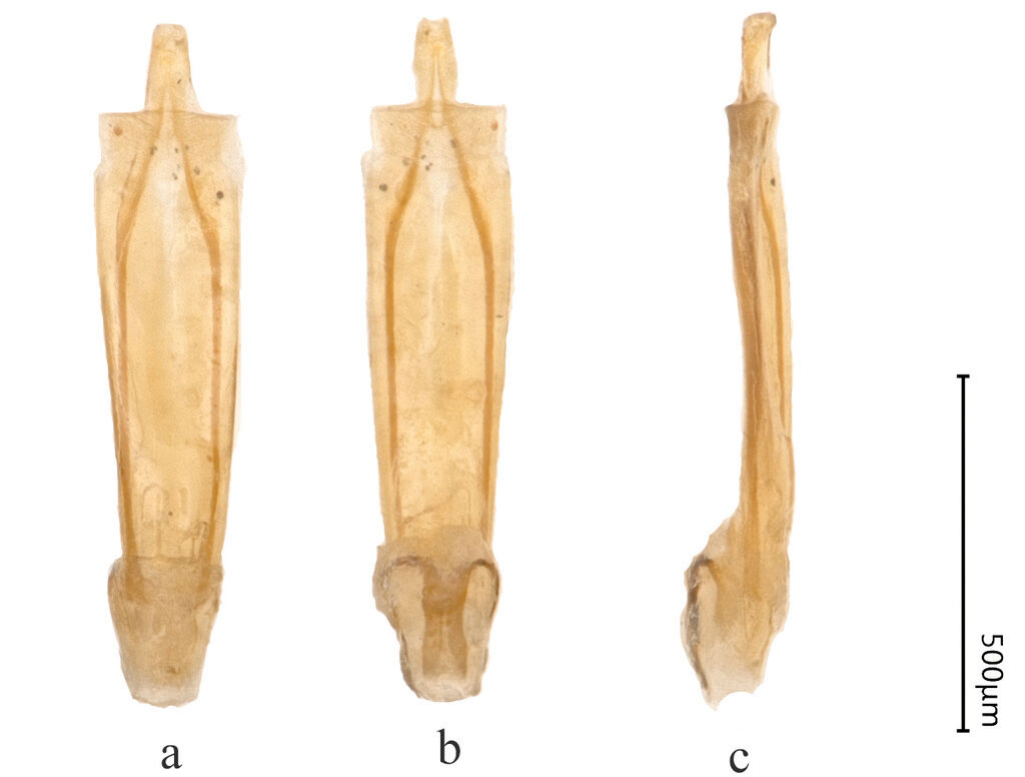
Male genitalia of Cantonius (Cantonius) anjiensis **sp. n.**: **a** dorsal view; **b** ventral view; **c** lateral view.

Male genitalia less sclerotised, parameres with apices rather widely emarginated (Fig. 2).

#### Diagnosis

##### Remarks

This new species is similar to C. (C.) klapperichi, C. (C.) megacephalus, C. (C.) obenbergeri and C. (C.) terryi by head distinctly wider than pronotum, but can be distinguished from all of these congeners by one of the following characters: 1) pronotal carinae slightly divergent anteriorly; 2) head relatively smaller, eyes larger; 3) body much more slender, 2.5–2.7 times as long as wide, while elytra 1.9–2.0 times as long as wide. It can also be diagnosed by the lateral margin of the pronotum strongly straight.

#### Etymology

The name of the species is derived from the type locality (Anji Mountains, Longnan County, Jiangxi Province) with reference to the species distribution.

#### Distribution

Known from type locality in the Anji Mountains (China: Jiangxi Province) at an elevation about 400 m.

#### Biology

Habitat: mostly along roadsides near to streams in the montane subtropical broadleaf evergreen forest (Fig. 3a).

Host plant: *Pleioblastusamarus*, Bambusoideae (Gramineae) (Fig. 3b).

### Cantonius (Procantonius) qiyunensis

Shi, Song & Xu sp. nov.

1C180F27-7D78-5AFB-B491-3202FC10287A

0524C1BB-7F0E-48C7-9E24-2E16A01E6972

#### Materials

- **Type status:** Holotype. **Occurrence:** catalogNumber: SXD2021001♂; sex: male; occurrenceID: EAB880CE-E012-5BEF-ADB2-7FE24EB6D016; **Location:** country: China; stateProvince: Jiangxi Province; county: Chongyi County; municipality: Ganzhou City; locality: Qiyun mountains; verbatimElevation: 630 m; locationRemarks: label: Qiyun mountains, Chongyi County, Ganzhou City, Jiangxi Province, elevation 630 m, feeding larvae in leaf-mines on *Bambusablumeana*, adults emerged on 05.Ⅲ.2021, Jia-Sheng Xu leg.; **Record Level:** institutionCode: LGNU
- **Type status:** Paratype. **Occurrence:** catalogNumber: SXD2021006♂, SXD2021007♂, SXD2021008♂; sex: 3 male; occurrenceID: 1E7F8F52-AFF4-5719-B22D-7F17E9D40EBF; **Location:** locationRemarks: same label data as holotype; **Record Level:** institutionCode: CHTS, LGNU

#### Description

**Figure 4.**
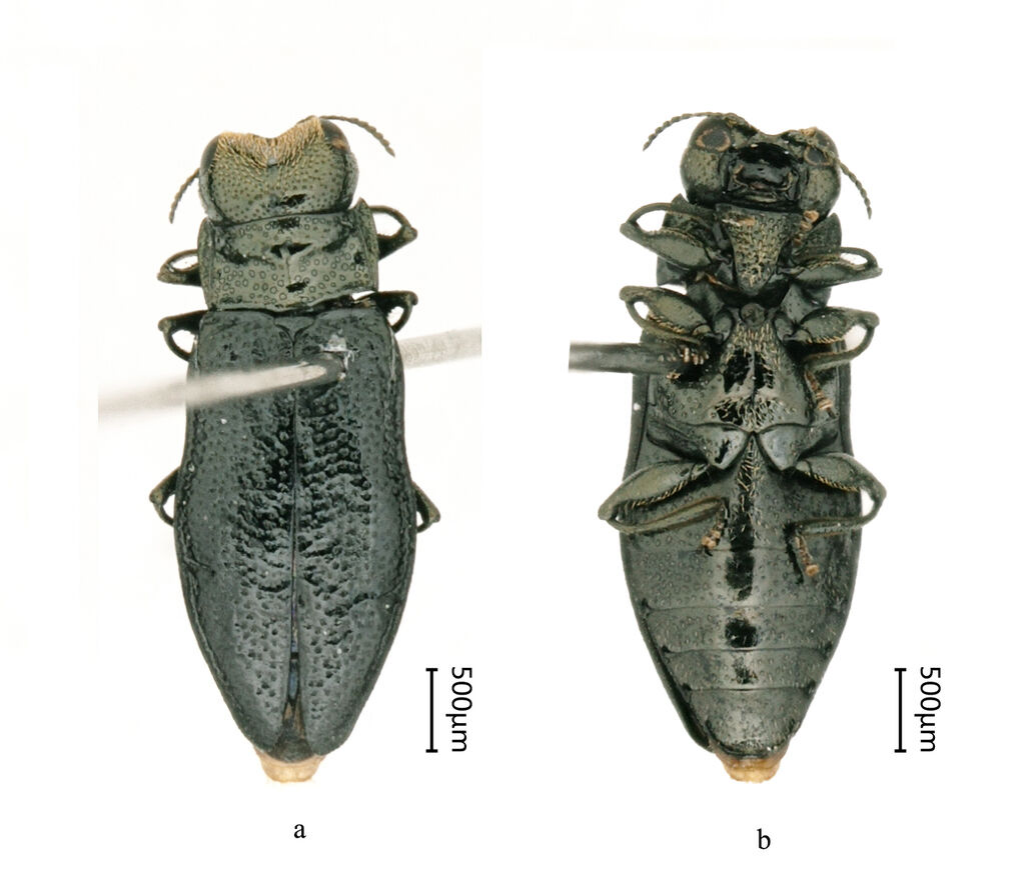
Habitus of Cantonius (Procantonius) qiyunensis **sp. n.**: **a** dorsal view of holotype male; **b** ventral view of holotype male.

Body length 3.4-3.9 mm, body width 1.2–1.5 mm. Body moderately narrow, 2.6–2.9 times as long as wide, moderately convex. Elytra blackish-bronze, but the head and pronotum bronze, rather lustrous, non-shagreened, with delicate, finely reticulate shagreenity only on the concave areas of pronotum and on ventral surface, with silky lustre (Fig. 4).

Head large and wide, about the same width as or slightly narrower than the pronotum, slightly angularly widened behind eyes, widest at mid-length between posterior margin of eyes and anterior margin of pronotum. Head depressed anteriorly. Eyes large, oval, moderately convex, not or slightly projecting beyond contour of head, almost as long as temples in dorsal view. Temples regularly arcuate. Surface with dense, simple, flat rounded punctures. Male with golden setae dense on frons and sparser on vertex. Antennae 11-segmented, serrate beginning with 6^th^ segment; 6^th^ segment slightly longer than wide, 7–10^th^ segments about as long as wide and 11^th^ segment longer than wide.

Pronotum 1.9–2.0 times as wide as long, with sides nearly straight or slightly irregularly and weakly arcuate, widest at anterior 1/3–1/4. Anterior margin strongly bisinuate, sharply concave on both sides, slightly raised in middle and mid-point much lower than anterior angles of pronotum. Posterior margin slightly bisinuate, basal lobe slightly projecting. Pronotum weakly convex. Lateral carinae nearly straight, directly to concaved part of anterior margin and not reaching anterior angles; posteriorly connected with raised posterior margin of pronotum, slightly inward from its posterior angles. Surface with scarce punctures. Flat punctures in depressed portions of disc. Scutellum large, transversely triangular.

Elytra 1.9–2.1 times as long as wide, much wider than pronotum and widest nearly at middle. Apices strongly separated, rounded, with several ill-defined teeth. Surface densely covered with coarse punctures and irregular coarse undulate transverse wrinkles.

Femora with a row of small teeth at inner side. Tibiae strongly arched. In female extended a little wider in posterior 1/3.

**Figure 5.**
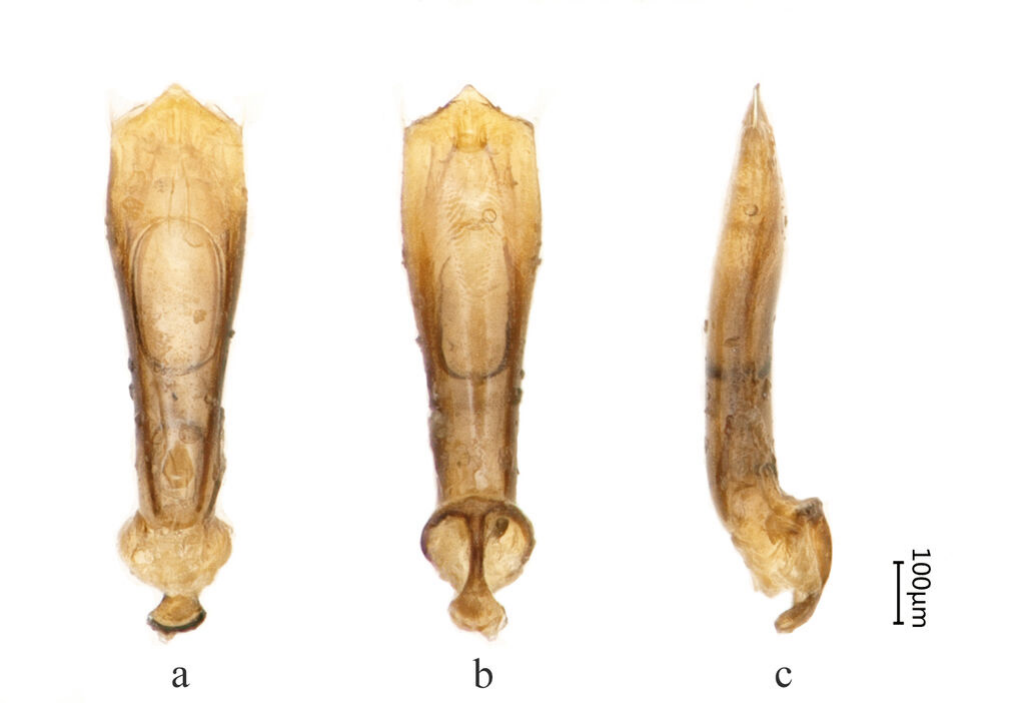
Male genitalia of Cantonius (Procantonius) qiyunensis **sp. n.**: **a** dorsal view; **b** ventral view; **c** lateral view.

Male genitalia strongly sclerotised, parameres triangular with rounded apices distally (Fig. 5).

Pupa in Fig. 6.

#### Diagnosis

##### Remarks

With the narrow body, 2.6–2.9 times as long as wide and elytra 1.9–2.1 times as long as wide, this new species resembles to C. (P.) jendeki Kalashian, 2004 and C. (P.) austrisinicus Kalashian, 2021. Temples almost regularly rounded can separate this species from C. (P.) austrisinicus and elytra widest nearly at middle can separate this species from C. (P.) jendeki. This new species can also be distinguished from all other congeners by the following characters: 1) head and pronotum bronze, but not blackish-bronze as the elytra; 2) anterior margin strongly bisinuate, the mid-point much lower than the anterior angles of the pronotum.

#### Etymology

The name of the species is derived from the type locality (Qiyun Mountains, Chongyi County, Jiangxi Province) with reference to the species distribution.

#### Distribution

Known from type locality in the Qiyun Mountains (China: Jiangxi Province) at an elevation about 700 m.

#### Biology

Habitat: mostly found in the alpine subtropical broadleaf evergreen forest in mountains along streams and roadsides (Fig. 7a).

Host plant: *Bambusablumeana*, Bambusoideae (Gramineae) (Fig. 7b).

Leaf mine: This species often have meandering shape mines. Larvae exist from late October to mid-March with only one bamboo leaf mine in Ganzhou. The mine appears as an amorphous blotch with a dark brown line in the early period (Fig. 8a). Then turn into a meandering thick line (Fig. 8c). The last instar larvae build pupal cell at the end of the leaf-mine and pupating inside (Fig. 8d). After biting the pupal epidermis (Fig. 8e, f), adult climb out and move around from March to May. Then they mate and lay eggs on the leaves of host plants which is easy to be found (Fig. 8b).

### Cantonius (Procantonius) austrisinicus

Kalashian, 2021

FAAFD30B-7EE0-50D9-8711-61A8690D5ABA

#### Materials

- **Type status:** Other material. **Occurrence:** sex: 19 males, 9 females; occurrenceID: 70DEB1E4-F469-5A84-B6AB-B4E0BD19C957; **Location:** country: China; stateProvince: Guangangxi Zhuang Autonomous Region; county: Shangsi County; municipality: Fangchenggang city; locality: Shiwandashan; locationRemarks: label: Shiwandashan, Shangsi County, Fangchenggang City, Guangxi Zhuang Autonomous Region. feeding larvae in leaf-mines on *Oligostachyumpaniculatum*, adults emerged 19.Ⅲ.2016–3.IV.2016, Xiao-Hua Dai leg.; **Record Level:** institutionCode: LGNU, CHTS

#### Description

Adult in Fig. 9.

Aedeagus in Fig. 10

#### Distribution

China (Guangxi, Guangdong).

#### Biology

**Figure 11.**
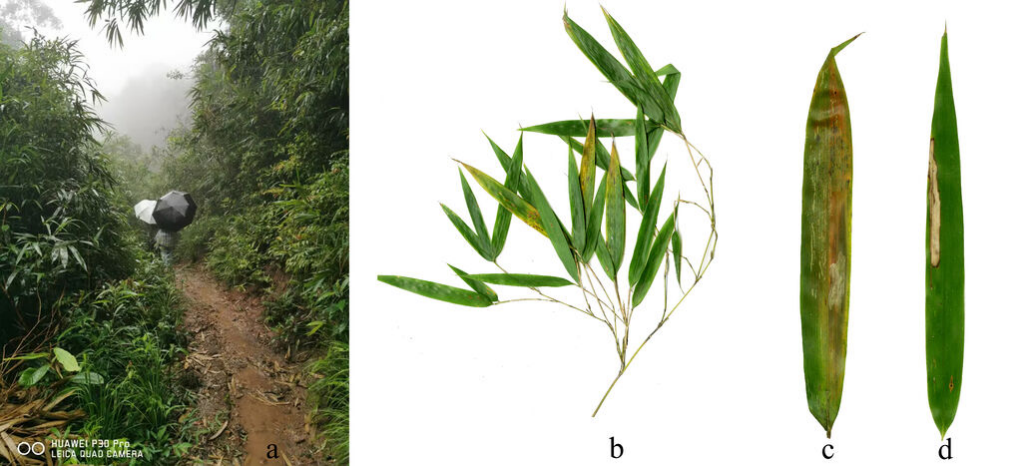
Biology of Cantonius (Procantonius) austrisinicus Kalashian, 2021: **a** habitat (photo by Xiao-Hua Dai); **b** host plant *Oligostachyumpaniculatum*; mine; **c** early period; **d** medium period.

Habitat and host plant: *Oligostachyumpaniculatum*, Bambusoideae (Gramineae) (Fig. 11).

## Discussion

The genus *Cantonius* is a tropical or subtropical taxon and has been reported mainly in Southeast Asia, central and southern China. Although some new species were published without any information of the host and biology of this genus. According to our collection and their feeding habits throughout the country, we found that *Cantonius* is commonly oligophagous and leaf-mining. All the three species mine leaves of Bambusoideae at the larval stage and the leaf-mining type of different *Cantonius* larvae is the same: the mine consists of irregular plaques or a thick line at the earliest period, winding forward along with the growth of the adult, then coarsening at the end period. This is a commonly winding mine type.

Typically, these insects have two generations every year, with the overwintering generation's adults emerging in the months of March through May. Its spawning grounds are easy to find since they mate and deposit eggs on the leaves of their host plants (Bambusoideae). The larvae mines into the leaves in search of the mesophyll tissues, and then spin a cocoon over the passage to cover itself. The emergence of the first generation of adults is usually completed in July and August of the same year. From September to October, the adults are free to move and again the same cycle is repeated. The adults of the second generation begin to mate and lay eggs in early November and the larvae overwinter until the adults appear in March to May of the following year.

In addition, our data and discussions with experts have shown that *Cantonius* species like wet, dense bamboo areas that are close to streams or other water bodies with low direct sunlight. *Cantonius* adults and larvae thrive in the damp, dark conditions provided by Bambusoideae leaves.

We hypothesize that *Cantonius* species may be oligophagous and only consume corresponding bamboo genera or even species based on the facts that the majority of species are narrowly distributed (with the exception of *C.austrisinicus* and *C.nitidifrons*) and that as many as three species have been found in the Wuyi Mountains without host data. Due to the abundance of bamboo resources in China as well as their association with various ecosystems and the physical obstacles that the mountains have built, there are likely many more new *Cantonius* species and records.

## Supplementary Material

XML Treatment for Cantonius (Cantonius) anjiensis

XML Treatment for Cantonius (Procantonius) qiyunensis

XML Treatment for Cantonius (Procantonius) austrisinicus

## Figures and Tables

**Figure 3a.**
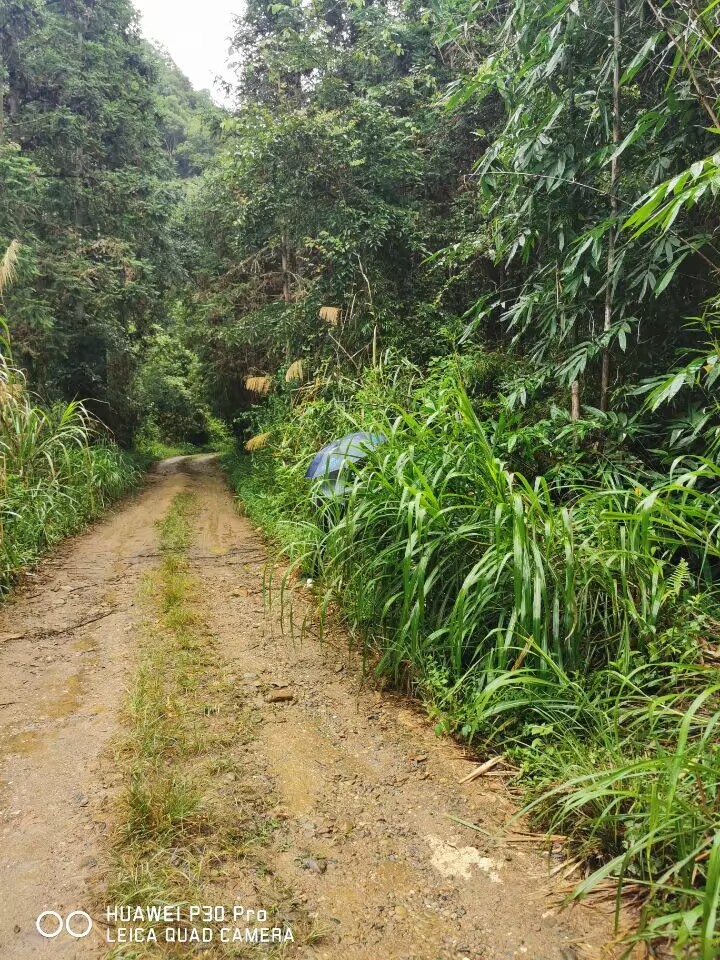


**Figure 3b.**
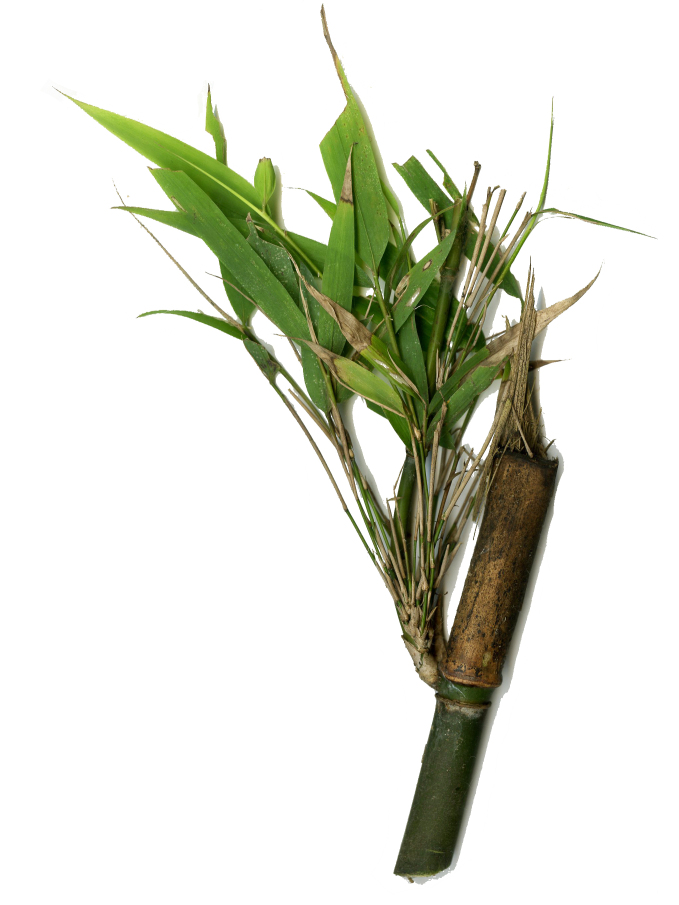


**Figure 6.**
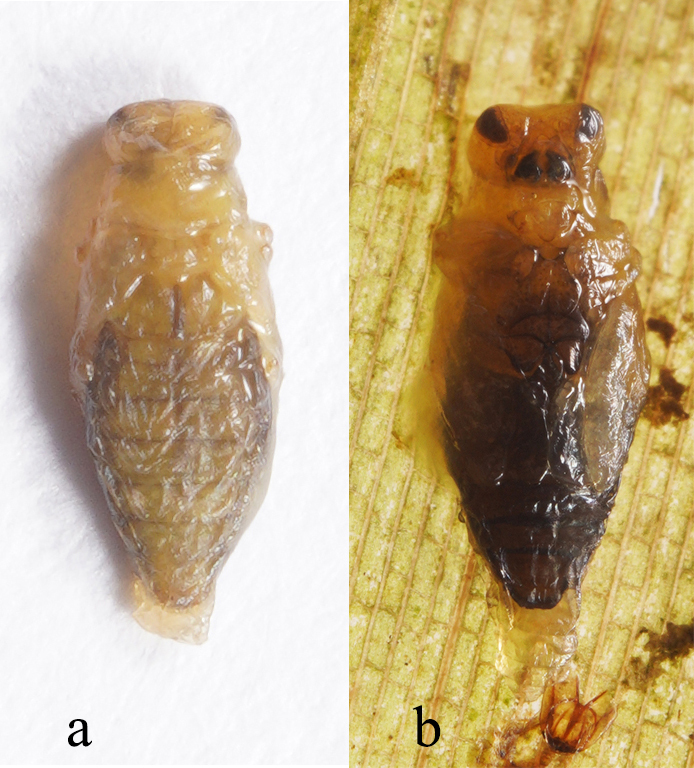
Pupa of Cantonius (Procantonius) qiyunensis **sp. n.**: **a** dorsal view; **b** ventral view.

**Figure 7a.**
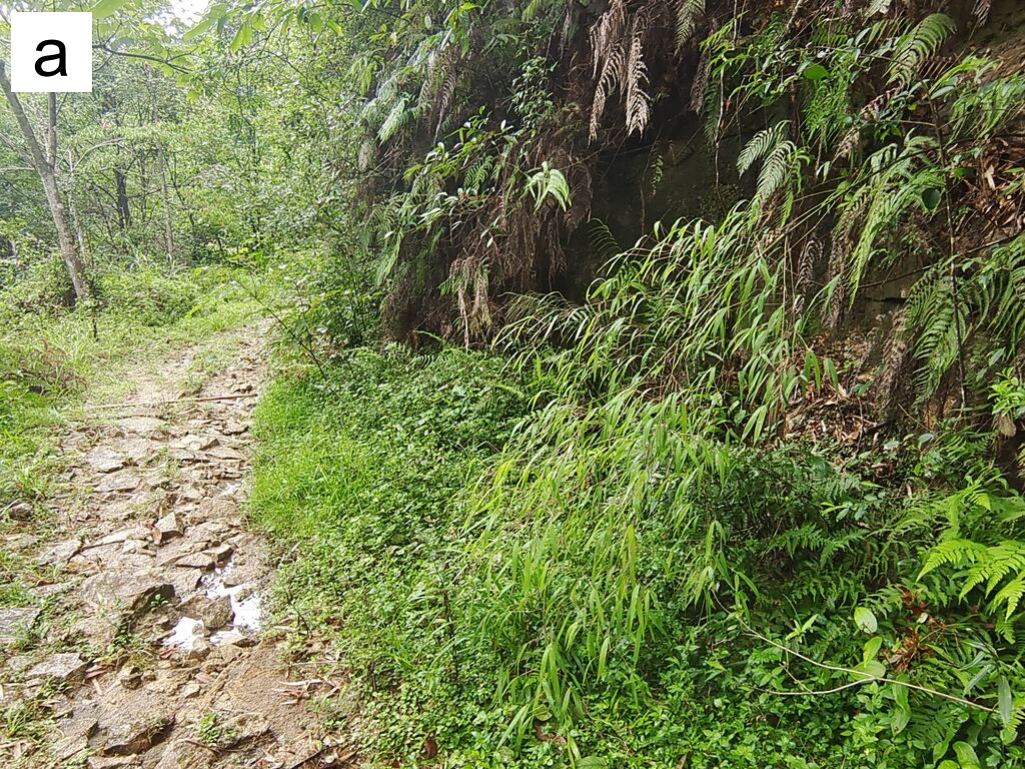


**Figure 7b.**
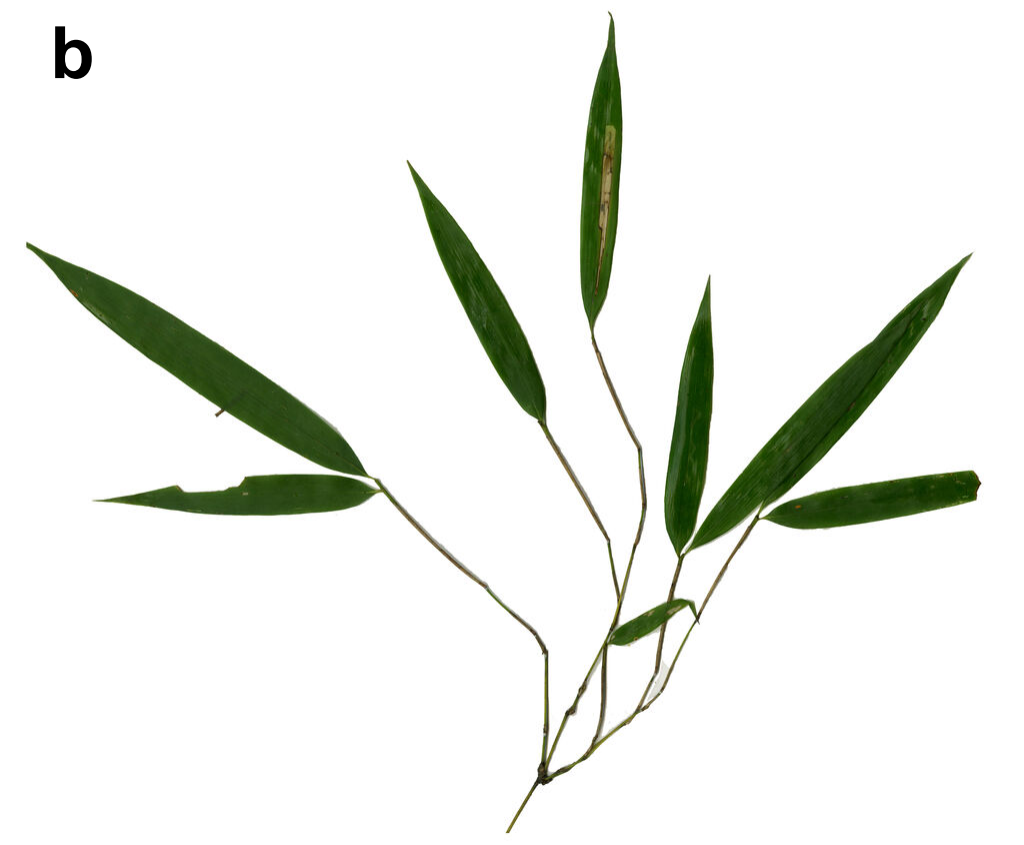


**Figure 8a.**
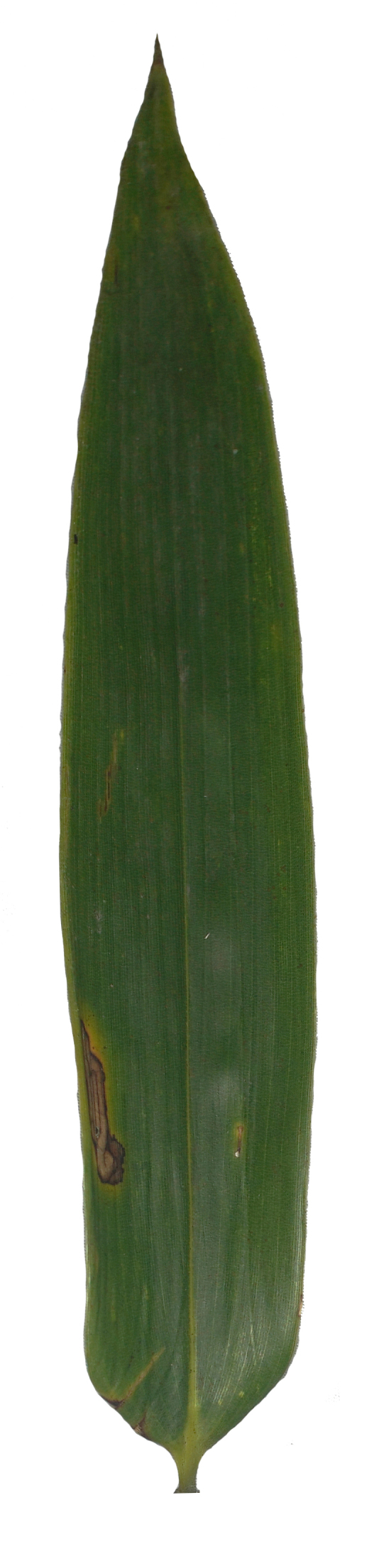


**Figure 8b.**
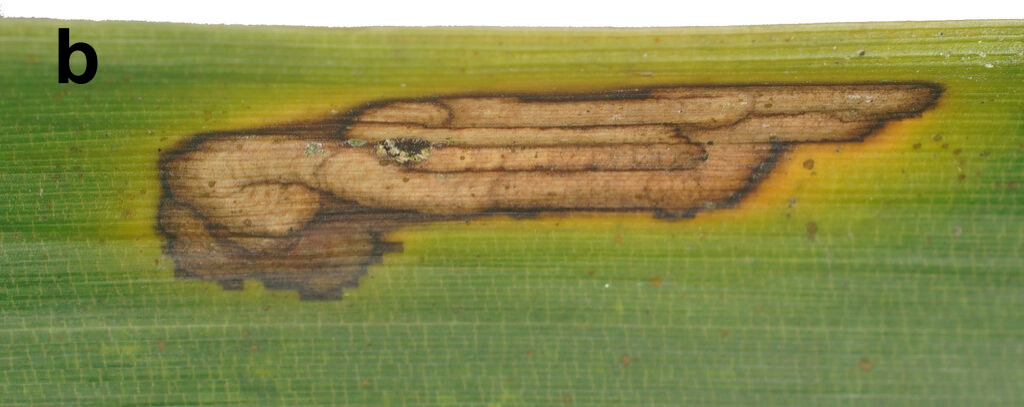


**Figure 8c.**
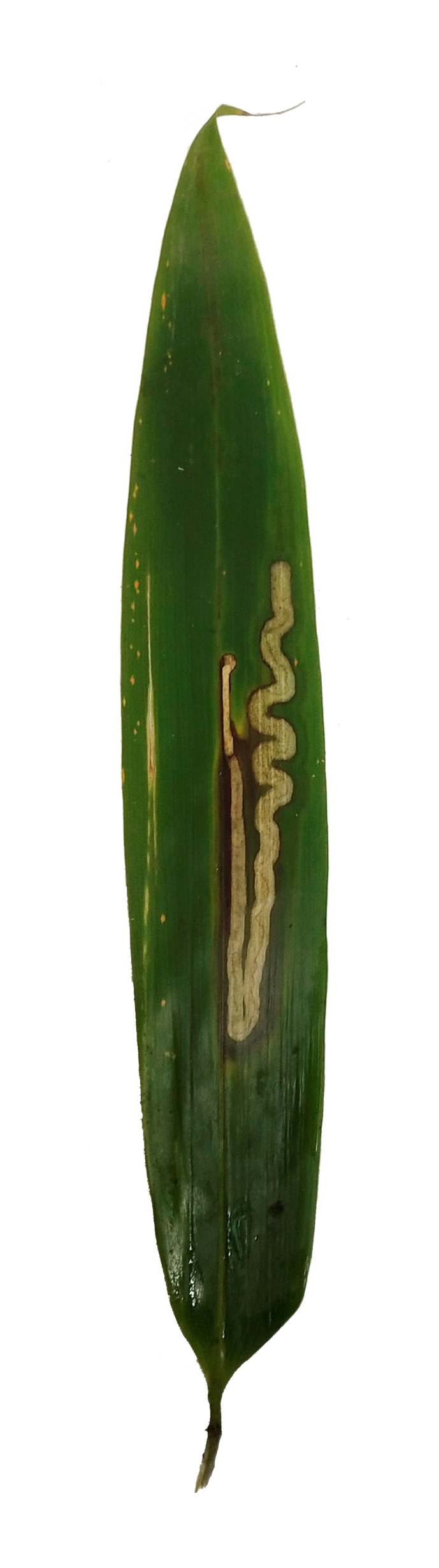


**Figure 8d.**
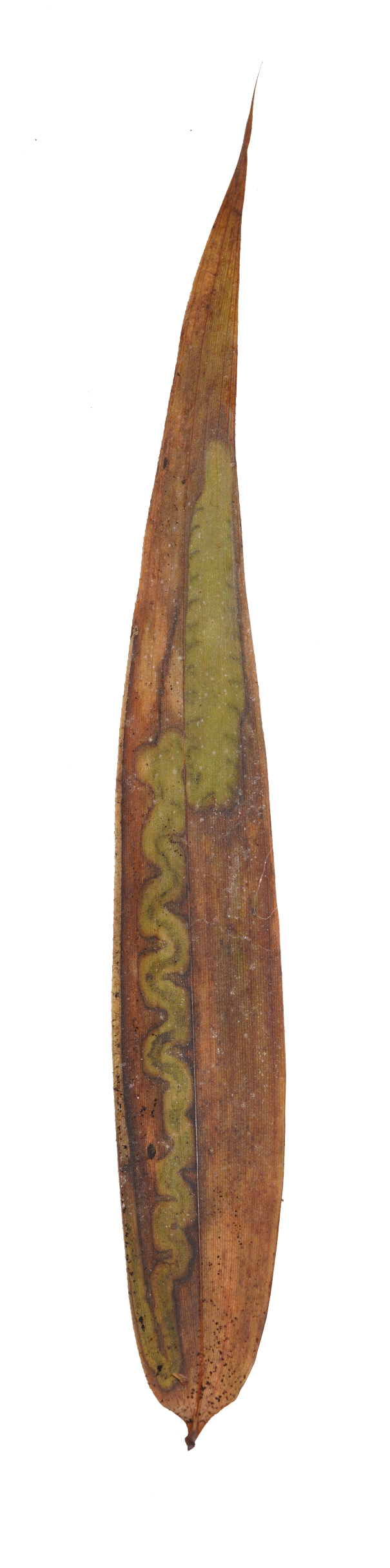


**Figure 8e.**
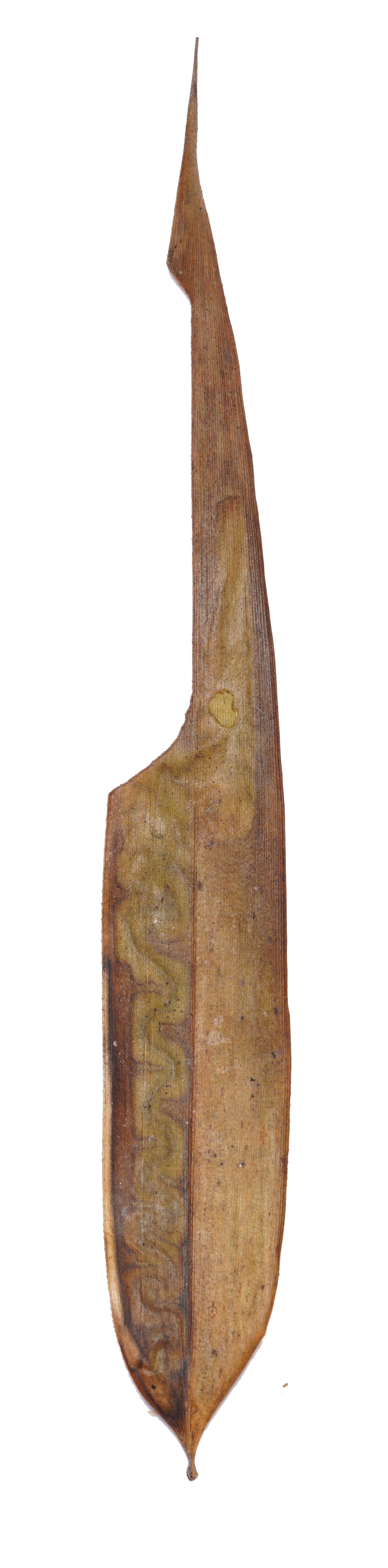


**Figure 8f.**
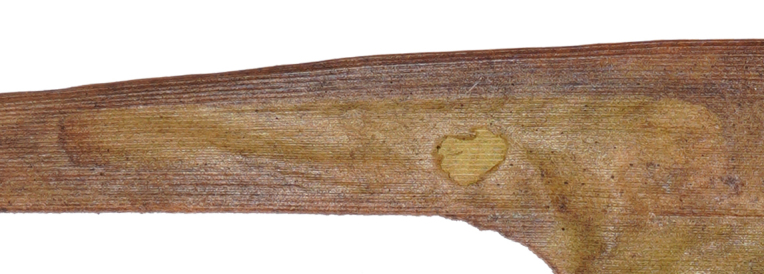


**Figure 9.**
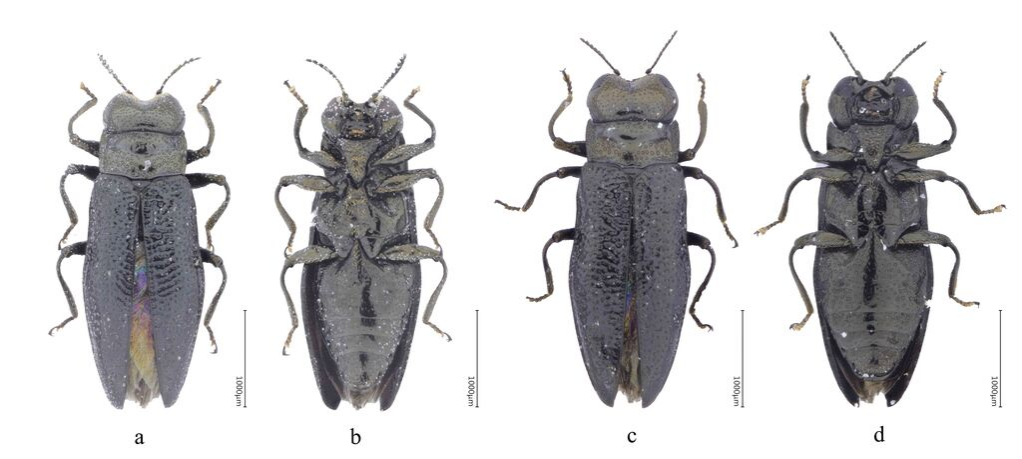
Habitus of Cantonius (Procantonius) austrisinicus Kalashian, 2021: **a** dorsal view of male; **b** ventral view of male; **c** dorsal view of female; **d** ventral view of female.

**Figure 10.**
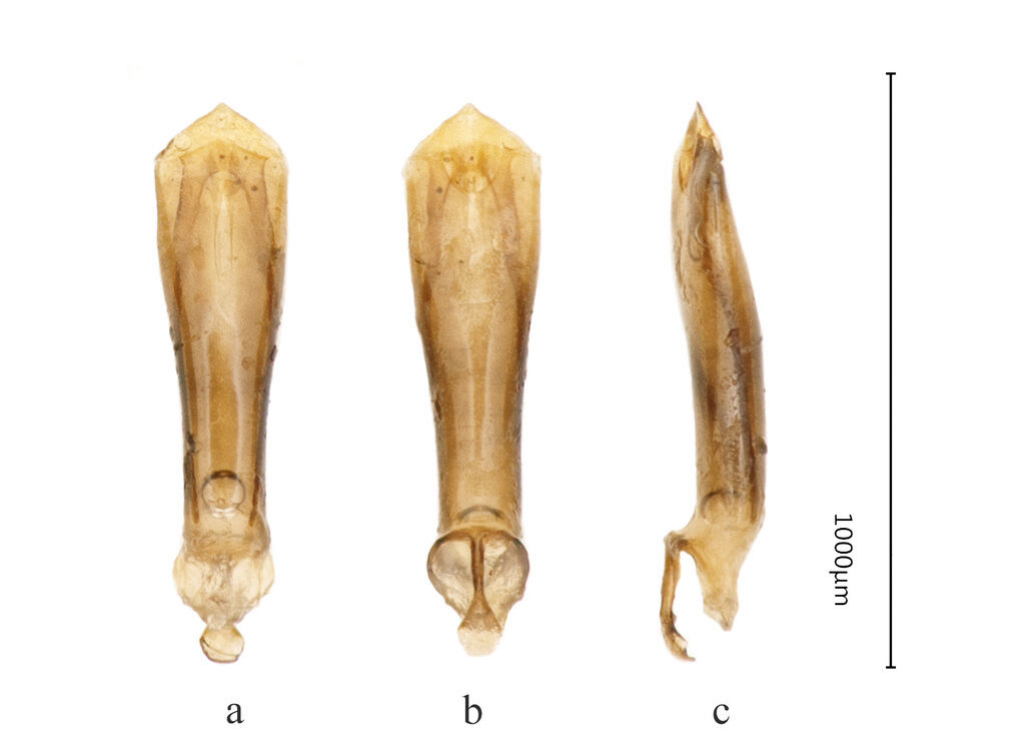
Male genitalia of Cantonius (Procantonius) austrisinicus Kalashian, 2021: **a** dorsal view; **b** ventral view; **c** lateral view.
